# Supplementary material for: Flexible combination of multiple diagnostic biomarkers to improve diagnostic accuracy
Source: BMC Med Res Methodol. 2015 Oct 31;15:94. doi: 10.1186/s12874-015-0085-z (PMC4628350; doi:10.1186/s12874-015-0085-z)
Supplement: Additional file 1 — The Appendix includes the proof of Proposition 1. (PDF 28.6 kb) [file 12874_2015_85_MOESM1_ESM.pdf]

Appendix for ”Flexible combination of multiple diagnostic biomarkers to improve diagnostic accuracy” by Xu, Rong, Fang and Wang

Proof of Proposition 1. Since $L_\delta(u) = L_{01}(u) + \delta^{-1}(\delta - u)I(0 \le u \le \delta)$, we have

$$
\begin{aligned}
E\Big(w(Y,\mathbf{Z})L_\delta(Y(g(\mathbf{X}) - c(\mathbf{Z}))\Big) &= E\Big(w(Y,\mathbf{Z})L_{01}(Y(g(\mathbf{X}) - c(\mathbf{Z}))\Big) \\
&\quad + E\Big(w(Y,\mathbf{Z})\frac{\delta - Y(g(\mathbf{X}) - c(\mathbf{Z}))}{\delta} I(0 \le Y(g(\mathbf{X}) - c(\mathbf{Z})) \le \delta)\Big).
\end{aligned} \tag{1}
$$

Note that $E\big(w(Y,\mathbf{Z})\frac{\delta - Y(g(\mathbf{X})-c(\mathbf{Z}))}{\delta} I(0 \le Y(g(\mathbf{X}) - c(\mathbf{Z})) \le \delta)\big)$ is decreasing in δ, and approaches 0 when $\delta \to 0$. Furthermore, for any given $\epsilon > 0$,

$$
\begin{aligned}
&E\big(w(Y,\mathbf{Z})L_{01}(Y(g(\mathbf{X}) - c(\mathbf{Z})))\big) - E\big(w(Y,\mathbf{Z})L_{01}(Y(g^*(\mathbf{X}) - c^*(\mathbf{Z}))\big) \\
=& \int_{\mathcal{D}_{g,c,0} \cap \mathcal{D}^c_{g^*,c^*,0}} \frac{\pi_{\mathbf{z}} - p_{\mathbf{z}}(\mathbf{x})}{\pi_{\mathbf{z}}(1-\pi_{\mathbf{z}})} f(\tilde{\mathbf{x}})d\tilde{\mathbf{x}} + \int_{\mathcal{D}^c_{g,c,0} \cap \mathcal{D}_{g^*,c^*,0}} \frac{p_{\mathbf{z}}(\mathbf{x}) - \pi_{\mathbf{z}}}{\pi_{\mathbf{z}}(1-\pi_{\mathbf{z}})} f(\tilde{\mathbf{x}})d\tilde{\mathbf{x}} \\
\ge& \int_{\mathcal{D}_{g,c,\epsilon} \cap \mathcal{D}^c_{g^*,c^*,\epsilon}} \frac{\pi_{\mathbf{z}} - p_{\mathbf{z}}(\mathbf{x})}{\pi_{\mathbf{z}}(1-\pi_{\mathbf{z}})} f(\tilde{\mathbf{x}})d\tilde{\mathbf{x}} + \int_{\mathcal{D}^c_{g,c,\epsilon} \cap \mathcal{D}_{g^*,c^*,\epsilon}} \frac{p_{\mathbf{z}}(\mathbf{x}) - \pi_{\mathbf{z}}}{\pi_{\mathbf{z}}(1-\pi_{\mathbf{z}})} f(\tilde{\mathbf{x}})d\tilde{\mathbf{x}},
\end{aligned} \tag{2}
$$

where $f(\tilde{\mathbf{x}})$ denotes the density function of $\tilde{\mathbf{x}}$. By **(??)**, we have

$$
\begin{cases}
\pi_{\mathbf{z}} - p_{\mathbf{z}}(\mathbf{x}) > \epsilon, & \text{if } \tilde{\mathbf{x}} \in \mathcal{D}_{g,c,\epsilon} \cap \mathcal{D}^c_{g^*,c^*,\epsilon}; \\
p_{\mathbf{z}}(\mathbf{x}) - \pi > \epsilon, & \text{if } \tilde{\mathbf{x}} \in \mathcal{D}^c_{g,c,\epsilon} \cap \mathcal{D}_{g^*,c^*,\epsilon}.
\end{cases}
$$

Therefore,

$$
E\big(w(Y,\mathbf{Z})L_{01}(Y(g^*_\delta(\mathbf{X}) - c^*_\delta(\mathbf{Z})))\big) - E\big(w(Y,\mathbf{Z})L_{01}(Y(g^*(\mathbf{X}) - c^*(\mathbf{Z})) > \epsilon Pr\left(\mathcal{D}_{g^*_\delta,c^*_\delta,\epsilon} \triangle \mathcal{D}_{g^*,c^*,\epsilon}\right).
$$

By the fact that $E\Big(w(Y,\mathbf{Z})L_\delta(Y(g^*_\delta(\mathbf{X}) - c^*_\delta(\mathbf{Z}))\Big) \le E\Big(w(Y,\mathbf{Z})L_\delta(Y(g^*(\mathbf{X}) - c^*(\mathbf{Z}))\Big)$, we have

$$
\begin{aligned}
&E\big(w(Y,\mathbf{Z})L_{01}(Y(g^*_\delta(\mathbf{X}) - c^*_\delta(\mathbf{Z})))\big) - E\big(w(Y,\mathbf{Z})L_{01}(Y(g^*(\mathbf{X}) - c^*(\mathbf{Z})))\big) \\
&\quad \le E\Big(w(Y,\mathbf{Z})\frac{\delta - Y(g^*(\mathbf{X}) - c^*(\mathbf{Z}))}{\delta} I(0 \le Y(g^*(\mathbf{X}) - c^*(\mathbf{Z})) \le \delta)\Big).
\end{aligned} \tag{3}
$$

It follows immediately that $Pr\left(\mathcal{D}_{g^*_\delta,c^*_\delta,\epsilon} \triangle \mathcal{D}_{g^*,c^*,\epsilon}\right) \to 0$ as $\delta \to 0$. □
